# Laparoscopic Diagnosis and Laparoscopic Hyperthermic Intraoperative Intraperitoneal Chemotherapy for Pseudomyxoma Peritonei Detected by CT Examination

**DOI:** 10.1155/2012/741202

**Published:** 2012-08-21

**Authors:** Masamitsu Hirano, Yutaka Yonemura, Emel Canbay, Masumi Ichinose, Tuyoshi Togawa, Takayuki Matsuda, Nobuyuki Takao, Akiyoshi Mizumoto

**Affiliations:** ^1^Department of Surgery, Kusatsu General Hospital, 1660 Yabase-Cho, Shiga, Kusatsu City 5258585, Japan; ^2^NPO Organization to Support Peritoneal Dissemination Treatment, 1-26 Harukimotomachi, Osaka, Kishiwada City 596-0032, Japan; ^3^Department of Surgery, Kishiwada TokushuKai Hospital, 4-27-1 Kamori-Cho, Osaka, Kishiwada City 596-8522, Japan; ^4^General Surgery Clinic, Derince Education and Research Hospital, 41900 Kocaeli, Turkey

## Abstract

*Background*. Patients with early stage of pseudomyxoma peritonei (PMP) are sometimes difficult to diagnose the primary sites and intraperitoneal spread of tumor and to perform a cytological study. *Methods*. Patients without a definitive diagnosis and with unknown extent of peritoneal spread of tumor underwent laparoscopy. Hyperthermic intraoperative intraperitoneal chemotherapy (HIPEC) was administered as part of the same intervention. The results of treatment were evaluated at the time of second-look laparotomy (SLL) as a subsequent intervention. *Results*. Eleven patients were managed by diagnostic laparoscopy followed by laparoscopic HIPEC (LHIPEC). The operation time of laparoscopic examination and LHIPEC was 177 ± 26 min (range 124–261 min). No intraoperative complication was experienced. The peritoneal carcinomatosis index (PCI) score by laparoscopic observation was 16.5 ± 6.4 (range 0–30). One patient with localized pseudomyxoma peritonei (PMP) mucocele did not received LHIPEC; the other 10 patients with peritoneal metastases (PM) were treated with LHIPEC. After LHIPEC, ascites disappeared in 2 cases and decreased in the amount in the other 8 cases. Nine patients underwent SLL and cytoreductive surgery (CRS) combined with HIPEC. The duration between LHIPEC and SLL ranged from 40 to 207 days (97 ± 40 days). The PCI at the SLL ranged from 4 to 27 (12.9 ± 7.1). The PCI at the time of SLL decreased as compared to PCI at the time of diagnostic laparotomy in 7 of 9 patients. Median follow-up period is 22 months (range 7–35). All 11 patients are alive. *Conclusion*. The early results suggest that laparoscopic diagnosis combined with LHIPEC is useful to determine the surgical treatment plan and reduce the tumor burden before definitive CRS at SLL.

## 1. Introduction

Pseudomyxoma peritonei (PMP) is an uncommon malignancy that is characterized with increases in abdominal girth due to massive accumulation of mucinous material throughout the peritoneal cavity [1, 2]. Frequency of PMP is reported as 1 to 2 per 1,000,000 population annually and 2 per 10,000 laparotomies. PMP develops in the peritoneal cavity by the perforation of mucinous material from malignant tumors from appendiceal (52%), ovarian (36%), colorectal (4%), and pancreatic (2%) origins [1, 2]. In the four largest reported series of 393 patients, immunohistochemistry techniques in women with both appendiceal and ovarian tumors favor an appendiceal primary in most cases [3].

Optimal treatment involves a combination of cytoreductive surgery (CRS) with hyperthermic intraperitoneal chemotherapy (HIPEC) [4, 5]. Computed tomography scanning (CT) and magnetic resonance imaging (MRI) are the optimal preoperative tools to determine the clinical stage [6]. However, the diagnosis of primary tumors and distribution of peritoneal carcinomatosis (PC) are sometimes difficult in patients with small amount of peritoneal nodules and ascites.

In Japan annual checkup for health using the measurement of serum tumor marker levels, ultrasonography (US) or CT is commonly performed as a mass screening. As a result the patients with appendiceal cystadenoma or cystadenocarcinoma are detected in an early stage by the US/CT and/or increased serum tumor marker levels. These patients usually show a small amount of ascites in the pelvis, elevation of carcinoembryonic antigen (CEA), and/or swelling of appendix with localized perforation. Determination of the histologic grade of the appendiceal primary tumor and the distribution of the intraperitoneal mucinous tumor is essential to planning treatment in this group of patients. It may assist in avoiding overtreatment in that patients with appendiceal neoplasm without perforation are not necessary to treat CRS and HIPEC, and appendectomy plus sampling of the regional lymph nodes is believed as an optimal treatment [7].

In addition, the preoperative knowledge of tumor whether it is low-grade or high-grade mucinous adenocarcinoma is important to make a surgical treatment plan and to expect the prognosis of the patient.

For the patients who are suggested to have appendiceal neoplasm with small amount of PM or without perforation, we studied the effectiveness of diagnostic laparoscopy combined with HIPEC at the same intervention (LHIPEC) as an adjunct to the management of PMP.

## 2. Patients and Methods

From April 2009 to January 2012, 125 patients with appendiceal neoplasm were treated at the Peritoneal Surface Malignancy Center of the Kusatsu General Hospital. Among them, eleven patients without a determined diagnosis were referred to the center for further examination, because they have no definitive diagnosis or no information regarding the peritoneal distribution of the tumor (Table 1). Radiologically, they had small amount of extraappendiceal mucinous appendiceal ascites thought to be a localized pseudomyxoma peritonei. On CT examination, they had swollen appendix and small amount of ascites in the pelvis, but no omental cake nor large mass in the peritoneal cavity. All 11 patients did not have a definitive diagnosis, had received operation, but were suspected to have appendiceal neoplasm with mucinous ascites.

**Figure 1:**
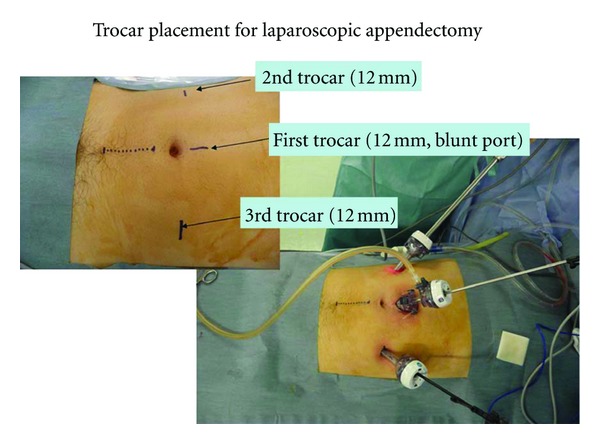


All patients received diagnostic laparoscopy (Figure 1). We used a 3-port configuration. A 12 mm blunt port was placed from the 2 cm longitudinal incision above the umbilicus. A second trocar (12 mm) was placed in the right upper quadrant, followed by a third trocar (12 mm) in the left lower quadrant. A 5 mm trocar was added if necessary in the left upper quadrant. The suction cannula was then used to evacuate the thick mucinous ascites, and samples were obtained for microbiologic cultures and cytology. Biopsy specimens were routinely obtained from peritoneum, omentum, and ovary. Spreading of the tumor in the entire abdominal cavity was evaluated using the peritoneal carcinomatosis index (PCI) based on the regions involved in the abdominal cavity and the sizes of the neoplastic nodules [3].

**Figure 2:**
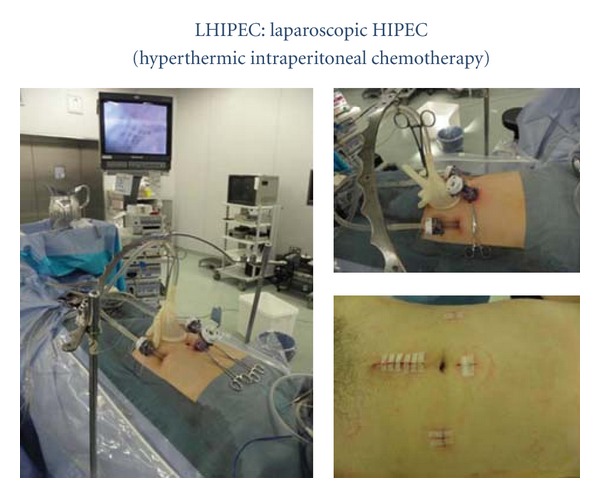


Appendectomy was performed, and the appendectomy specimen was evaluated histopathologically by frozen section. Following confirmation of the diagnosis, a longitudinal 5 cm midline incision was made to the lower abdomen for open laparotomy. Three drainage tubes (2 inlet tubes, 1 outlet tube) were placed for LHIPEC. The inside of the abdominal cavity was washed out with 10 liters of physiological saline solution to remove mucinous ascites, and hyperthermic intraperitoneal chemotherapy (HIPEC) was performed at 42°C to 43°C for 60 minutes adding 3 to 5 liters of the saline solution including 20 mg of mitomycin C and 100 mg of cisplatin (Figure 2).

## 3. Results

In all 11 patients the ascites was found on CT accompanied by a swelling, a cystic mass, or thickening of the appendiceal wall. The mean maximum diameter of the appendix was 27.1 mm (range 7–64 mm), and three of them showed calcification. Serum CEA levels ranged from 2.1 to 87.9 ng/mL (25.6 ± 19.1); 7 patients showed a higher CEA and CA19-9 levels than normal (Table 1).

The operation time of laparoscopic examination and HIPEC was 177.2 ± 25.8 minutes (range, 124–261 minutes); blood loss was always less than 20 mL (Table 2). No intraoperative complication was experienced. The PCI score by laparoscopic observation was 16.5 ± 6.4 (range 0–30). After operation, two patients developed renal dysfunction, but it improved after the 7th postoperative day.

Ascites examined by CT disappeared in 2 cases and decreased in amount in the other 8 cases within 2 months after LHIPEC. Serum CEA levels of all cases decreased after LHIPEC and became in the normal range in 5 cases.

Nine patients underwent SLL and CRS combined with HIPEC. The duration between LHIPEC and SLL ranged from 40 to 207 days (97.4 ± 40.6 days). PCI at the SLL ranged from 4 to 27 (12.9 ± 7.1); PCI at the time of SLL had decreased as compared to that recorded at the time of LHIPEC in 7 of 9 patients. However, PCI of 2 patients (case 3, 11) in SLL was higher than those at LHIPEC. Median follow-up period is 22 months (range 7–35 months). All 11 patients are alive. One patient developed port site recurrence 10 months after LHIPEC. He underwent redo surgery and had small recurrence nodules on the spleen, right paracolic gutter, and port site on the right lower abdomen. All the three sites of progressive disease were completely removed (Table 2).

## 4. Discussion

In general, PMP is treated with conventional open abdominal surgical procedure, because the metastases have already spread into the whole abdominal and pelvic space. The patients with advanced PMP have massive mucinous ascites, and the diagnosis can be made by CT, MRI, serum CEA levels, and cytological examination with abdominal paracentesis [6]. However, these modalities may not provide enough information to make a definitive treatment plan for the patient [1, 9]. Diagnostic laparoscopy provides a wide view of the whole abdominal cavity without a large incision [8, 10]. In addition, a pathological diagnosis from nodules on the peritoneal surface can be made during laparoscopy. The primary lesions of PMP are usually from a borderline malignancy of the appendix. The primary site is ovary, colon, and pancreas in 30% of all PMP cases [11]. The laparoscopic exploration is helpful for the definitive diagnosis of PMP in patients in whom a primary disease site is not identified [10].

In patients with ascites seen on radiologic studies who need a definitive diagnosis, laparoscopy is considered as a good diagnostic modality to determine the primary sites, dissemination of tumors, and histological diagnosis. Raj et al. and Kotani et al. [8, 12] performed laparoscopic-assisted surgery for patients with PMP, and they concluded that laparoscopic surgery allowed a wide area of observation within the abdominal cavity. Furthermore, a histological diagnosis of the appendiceal and ovarian tumor can be made after laparoscopic resection. The appendix should be examined histologically using immunohistochemistry, and the primary site of PMP and the histological grade can be obtained before SLL [13].

In the present study, PCI at SLL in 7 of 9 patients had decreased compared to that recorded at LHIPEC. Accordingly, LHIPEC may be effective to decrease PCI. In addition, serum CEA levels after LHIPEC significantly decreased as compared with those at LHIPEC. Since serum tumor marker levels correlate with the tumor burden [14], LHIPEC can decrease tumor volume. The present study suggests that laparoscopic diagnosis combined with LHIPEC is useful to determine the proper treatment and reduce the tumor burden before CRS at SLL.

## Figures and Tables

**Table 1:** Profiles of patients.

Patient no.	Age	Sex	Chief complaints (initial symptoms)	Preoperative diagnosis	Diameter of appendix (mm)	CEA (<6.0 ng/mL)	CA19-9 (<37.0 U/mL)
1	59	Female	Abdominal distension	Suspicious PMP	18	24.5	143.9
2	54	Male	Lower abdominal pain	Suspicious PMP	64	11.3	79.2
3	49	Female	Lower abdominal mass	Suspicious PMP	32	5.1	49.2
4	54	Male	None (serum CEA elevation)	Appendiceal tumor	7	54	17.4
5	44	Female	Abdominal pain	Suspicious PMP	35	87.9	540.4
6	58	Female	None (abnormal findings on US)	Suspicious PMP	14	26.1	148.4
7	67	Male	Right inguinal mass and pain	Suspicious PMP	50	4.5	7.9
8	56	Female	None (abnormal findings on US)	Appendiceal tumor	22	3.1	36.6
9	63	Female	Lower abdominal distension	Suspicious PMP	25	2.1	18.5
10	42	Male	None (abnormal findings on CT)	Suspicious PMP	19	46.5	137.6
11	60	Female	None (abnormal findings on US)	Suspicious PMP	11	33.4	91.9

**Table 2:** Results of laparoscopic operation and secondary look laparotomy (cytoreductive surgery + HIPEC).

Patient no.	Procedures	Operative time (min)	PCI at LHIPEC	Hospital stay after ope. (days)	Postoperative complications	Postoperative serum CEA	Changes of ascites on CT	Period from LHIPEC to SLL (days)	PCI at SLL	Follow-up period (months)
1	LAp + LHIPEC	124	25	16	(−)	Normal	↓	165	5	35
2	LAp + LHIPEC	261	14	13	(−)	Normal	↓	55	4	33
3	LAp + LHIPEC	153	12	14	RDF	Normal	↓	207	23	26
4	LAp + LHIPEC	160	12	4	(−)	Decreased	Disappeared	42	8	29
5	LAp + LHIPEC	151	30	15	(−)	Decreased	↓	115	27	23
6	LAp + LHIPEC	189	25	13	(−)	Decreased	↓	84	11	23
7	LAp + LHIPEC	201	11	9	RDF	Normal	↓	94	7	18
8	LAp	148	0	6	(−)	(−)	(−)	(−)	(−)	(−)
9	LAp + LHIPEC	201	12	11	(−)	Normal	Disappeared	40	7	7
10	LAp + LHIPEC	189	21	8	(−)	Decreased	↓	(−)	(−)	(−)
11	LAp + LHIPEC	172	20	9	(−)	Elevated	↓	50	24	7

LAp: laparoscopic appendectomy.

LHIPEC: laparoscopic hyperthermic intraperitoneal chemotherapy.

PCI: peritoneal carcinomatosis index.

SLL: second look laparotomy.

RDF: renal dysfunction improved within 1 week.

No.  4: port site recurrence was revealed 10 months after LP1PEC.

No.  8: localized PMP (mucocele).
